# An event-driven approach for studying gene block evolution in bacteria

**DOI:** 10.1093/bioinformatics/btv128

**Published:** 2015-02-25

**Authors:** David C. Ream, Asma R. Bankapur, Iddo Friedberg

**Affiliations:** ^1^Department of Microbiology, Miami University, Oxford, OH, USA and ^2^Department of Computer Science and Software Engineering, Miami University, Oxford, OH, USA

## Abstract

**Motivation:** Gene blocks are genes co-located on the chromosome. In many cases, gene blocks are conserved between bacterial species, sometimes as operons, when genes are co-transcribed. The conservation is rarely absolute: gene loss, gain, duplication, block splitting and block fusion are frequently observed. An open question in bacterial molecular evolution is that of the formation and breakup of gene blocks, for which several models have been proposed. These models, however, are not generally applicable to all types of gene blocks, and consequently cannot be used to broadly compare and study gene block evolution. To address this problem, we introduce an event-based method for tracking gene block evolution in bacteria.

**Results:** We show here that the evolution of gene blocks in proteobacteria can be described by a small set of events. Those include the insertion of genes into, or the splitting of genes out of a gene block, gene loss, and gene duplication. We show how the event-based method of gene block evolution allows us to determine the evolutionary rateand may be used to trace the ancestral states of their formation. We conclude that the event-based method can be used to help us understand the formation of these important bacterial genomic structures.

**Availability and implementation:** The software is available under GPLv3 license on http://github.com/reamdc1/gene_block_evolution.git. Supplementary online material: http://iddo-friedberg.net/operon-evolution

**Contact:** i.friedberg@miamioh.edu

**Supplementary information:** Supplementary data are available at *Bioinformatics* online.

## 1 Introduction

In bacterial and archaeal genomes, gene blocks are sequences of genes co-located on the chromosome, whose evolutionary conservation can be conservation of gene blocks can be strikingly apparent (Overbeek *et al.,* 2005; Pellegrini *et al.,* 1999) and has been used in phylogenetic and functional studies (Enault *et al.,* 2003; Overbeek *et al.,* 1999, 2014; Srinivasan *et al.,* 2005). Conservations across numerous taxa indicate that at least some conserved blocks are operons: a special case of gene blocks where the genes are co-transcribed to polycistronic mRNA and are often associated with a single function, such as a metabolic pathway or a protein complex. It is estimated that 5–50% of bacterial genes reside in operons (Cherry, 2003; Wolf *et al.,* 2001). Typically operons are under the control of one or more regulator proteins, which facilitate co-regulated transcription. From an evolutionary point of view, there are several questions that are asked about operons and gene blocks. How did these units evolve? What confers fitness upon genes in an operon or block structure as opposed to not being neighboring? Are certain operons more or less evolutionarily conserved in bacteria? What affects the conservation of the operon or gene block structure in different taxa?

Several models exist to explain gene block evolution (for more extensive reviews see Fondi et al., 2009; Martin and McInerney, 2009). One of the first models proposed for biopathway evolution is the Natal or Retrograde model which proposed that genes are arranged in blocks and operons due to tandem gene duplications derived from the depletion of metabolites in the environment (Horowitz, 1945). However, this model does not explain many operons which encode for proteins that are not homologous. Early *co-adaptation models* (reviewed in Stahl and Murray, 1966) applied to operons propose that neighboring genes into operons would lower the chances of co-adapted genes being separated by random recombination. However, orthologous replacements of operon genes have been observed, suggesting that preservation of co-localization of co-adapted alleles is not an exclusive reason for operons to form. The *coregulation model* is derived from the original definition of an operon: that the neighboring operon genes is due to the increased benefit of coregulation, providing an increased fitness for the population which has the operon (Price *et al.,* 2005). However, intermediate stages involving the cotranscription of non-beneficial genes cannot explain an incremental increase in fitness. The *selfish operon* model (Lawrence and Roth, 1996) proposes that the formation of gene blocks in bacteria is mediated by transfer of DNA within and among taxa. The model proposes an increase in fitness for the constituent genes because it enables the transfer of functionally coupled genes that would otherwise not increase fitness if they were separate. Furthermore, the joining of genes into blocks and eventually operons is beneficial for the horizontal gene transfer (HGT) of weakly selected, functionally coupled genes. Thus, we expect to see a certain percentage of ‘genetic hitchhikers’: non-beneficial genes that are coupled to beneficial genes in the operon. It seems that the selfish operon model does account for the structure of some operons, but is not the only mechanism of operon construction. The main finding against the selfish operon model’s exclusivity is the much lower number of ‘hitchhiking’, non-essential genes than expected (Pál and Hurst, 2004). Price *et al.* proposed that operon evolution is being driven by selection on gene expression patterns, and they also found that although genes within operons are usually closely spaced, genes in highly expressed operons may be widely spaced because of regulatory fine-tuning by intervening sequences. This study was based on a comparative analysis of two genomes, but included extensive expression data (Price *et al.,* 2006). Another model is that of the *mosaic operons* (Omelchenko *et al.,* 2003). In this model, shuffling, disruption, and HGT play dominant parts in operon formation. Under this model, an operon is not a steady-state evolutionary entity, but rather a dynamic entity which continuously acquires or loses genes via HGT. In their study, Omelchenko *et al.* have shown that although some operons follow the selfish model, many do not, with HGT of individual genes into operons being quite common. Whole operon transfer was identified in ∼30% of the operons studied. Another 20% of the operons were identified as mosaic operons. However, the study does not attempt to further classify the different types of mosaic operons, but rather provides an in-depth study of some of them. A study of the *his* operon by Fani *et al.* (2005) has proposed a ‘piecewise’ model to operon evolution. The piecewise model suggests that the construction of the *his* operon is a sequential series of events starting from a scattered set of constituent genes. Other models include an adaptive life cycle of operons, which explains why they rarely evolve optimally (Price *et al.,* 2006). To explain the persistence of gene blocks and operons once they are formed, Fang *et al*. (2008) suggest that co-transcription, protein–protein interactions and functional coupling are a stabilizing force maintaining genomic neighborhoods (Fang *et al.,* 2008). One interesting study has done away with genes as the atomic unit of gene blocks, and instead investigated domain rearrangements to identify and classify conserved gene blocks (Pasek *et al.,* 2005). Another process suggested to contribute to the formation of gene blocks is that of strand replication bias (Rocha and Danchin, 2001). It should be noted that plasmids are also a platform that selects for and maintains gene blocks (González *et al.,* 2003); however, in this work we limited our study to chromosomal DNA.

Each of the operon evolution models presents a mechanism and fits a biological rationale to the observation that operons/gene blocks exist in extant taxa. However, these models do not readily allow us to quantify the changes between either operons/gene block or between different organisms. Moreover, more than one model can generally be applied to a chosen gene block and set of taxa. Therefore, there is a need to create a universally applicable method for charting gene block evolution, and quantifying its conservation across taxa. Having such a method on hand can help determine the specific evolutionary trajectory of any given gene block. Here we describe such a method, which, in conjunction with a novel phylogenetic visualization method, dubbed ‘phylomatrix’, enables the quick examination of gene block conservation across taxa.

## 2 Approach

We present a novel approach to investigate gene block evolution which we call the *event-driven method*. Our approach borrows from the model describing the evolution of DNA and protein sequences. The accepted model for sequence evolution posits two types of basic events leading to change over time in DNA or protein sequences: indels and mutations. Indels and mutations are assigned scores based on the frequency of their occurrence over time (Dayhoff, 1978; Henikoff and Henikoff, 1992). Given a pair of sequences, a typical hypothesis is posed as to whether they are homologs. The hypothesis is not rejected if we can show that these two sequences are similar with a reasonable statistical significance. In practical terms, the similarity between two sequences is ascertained if the cumulative score of indels and mutation events differentiating the sequences is above a certain threshold, determined by an appropriate null model, so that it can be stated that the sequences are significantly similar. Note that the smallest unit in which a change can happen is the nucleotide (DNA) or the amino-acid (protein).

**Fig. 1.**
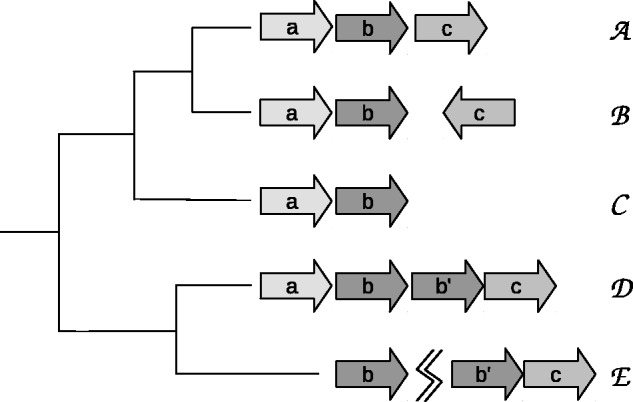
The event-driven approach for operon evolution. Species A−E are arranged in a phylogenetic species tree. A is a source taxon, with gene block A(a,b,c). In species B there is a strand reversal of the homolog Bc, which is treated as a split. The orthoblock in species C has a deletion of gene Cc when compared with species A or B. The orthoblock in D has a duplication of gene Bb in relation to taxon A. The orthoblock in species E has a split and a deletion of gene Ea

The event-driven method of gene block evolution describes evolutionary events that occur between gene blocks that are homologous between different bacterial species. The atomic unit of change is now the *gene* as a building-block of a gene block, rather than the nucleotide as the building block of a gene. This procedure is best explained by example. Suppose that genome A has neighboring genes A(a,b,c) in that order (In this annotation, upper case letters are the taxon, lower case letters are the genes). Genome B has homologs to those in A B(a,b) are neighboring, but Bc is located somewhere else in the chromosome, and reversed. As for genome C, *c* was deleted, so C(a,b) are neighboring. For the scenario described, we can say that there was a gene split event between A and B, and a gene deletion event between C and any of the other genomes for the gene block A(a,b,c). When the phylogenetic tree is known, these events can be placed on the tree. See full example in Figure 1.

If changes in gene blocks can be represented using a small set of events, the number and type of these events can be used to describe the evolutionary history of the gene block. Here we report on a set of 38 operons from *Escherichia coli* whose homologs we have examined across 33 taxa of proteobacteria. For these 38 operons and related orthologous gene blocks, we show an implementation of the event-driven approach to operon evolution.

## 3 Methods

We define the following concepts: **reference taxon** is a taxon where operons have been identified by experimental means. Here we use *E.coli* K-12 MG1655 as the reference taxon. We chose *E.coli* because it is expertly and comprehensively annotated in the RegulonDB database (Salgado *et al.,* 2006). **Neighboring genes**: two genes are considered neighboring if they are 500 nucleotides or fewer apart, and on the same strand. A **gene block** comprises no fewer than two open reading frames, or ORFs which are neighboring. An **event** is a change in the gene block between any two species with homologous gene blocks. **Orthoblocks** (gene blocks that are orthologous) are defined as follows: two organisms have orthoblocks when each organism must have at least two neighboring genes that are homologous to genes in a gene block in the reference taxon’s genome. Genes are considered homologous if their pairwise BLAST e-value is 10^−^^10^ or less. Relying on a strict BLAST threshold may exclude homologous proteins whose sequence similarity is not high (false negatives). However, this strategy will rarely include proteins with a different function (false positives). This rigorous threshold was chosen with the primary goal of minimizing false positives when inferring function by similarity.

### 3.1 Evolutionary events

Next we define events that we use to examine changes in gene block structure between different bacterial taxa relative to *E.coli*. We chose a set of target taxa with known phylogenetic relationships. The genomes of the target taxa were searched for homologous blocks to the operons found in *E.coli*. The operons we chose were selected based on the following criteria: (i) all the genes were protein coding; (ii) for all blocks chosen, the co-transcription was experimentally determined in *E.coli*; (iii) each operon comprised at least five genes; (iv) each operon has orthoblocks in at least nine other genomes. Using these filtering criteria, and RegulonDB’s annotation of *E.coli* as our reference taxon, we compiled 38 operons for this study. See Supplementary Material, Table S1 for a full list of operons, and Supplementary Table S3 for a list of taxa used.

We define the following pairwise events between orthoblocks from different taxons:

1. **Splits** If two genes in one taxon are neighboring and their homologs in the other taxon are not, then that is defined as a single *split event*. The distance is the minimal number of split events identified between the compared genomes.
2. **Deletions** A gene exists in the operon in the one taxon, but its homolog cannot be found in an orthoblock in another taxon. Note that the definition of homolog, *e*-value 10^−^^10^ is strict, and may result in false negatives. The *deletion distance* is the number of deletion events identified between the compared target genomes.
3. **Duplications** A duplication event is defined as having gene *j* in a gene block in the source genome, and homologous genes (j′,j″) in the homologous block in the target genome. The *duplication distance* is the number of duplication events counted between the source and target genomes. The duplication has to occur in a gene block to be tallied.

Other events were examined too: rearrangement of genes, genes moving to another strand, fusion and fission of ORFs. These event types correlated strongly with one or more of the three event types listed above, and were therefore discarded. Fusion/fission of ORFs was rare in our data set, so this event type was discarded as well.

The event-driven method does not account for HGT, which is suggested as a common mechanism for transferring neighboring genes (Lawrence and Roth, 1996). However, we have not yet incorporated HGT into our model. We have tried using AlienHunter (Langille *et al.,* 2008), and the IslandViewer (Langille and Brinkman, 2009) suite to detect HGT events in our data. Given that the taxa we are analyzing are closely related, these softwares were unable to detect HGT events.

### 3.2 Different conservation rates for gene blocks in proteobacteria

#### 3.2.1 Determining orthology

To trace the events that affect genes in gene blocks, it is necessary to determine which genes are orthologous between any two taxa when more than two possible homolog pairings may exist. The problem may be stated as follows: given a gene *g* in genome *A*, and a set of homologs to *g* in genome *B*, $H_{g}^{B}=\{g_{1},g_{2},...,g_{n}\}$, which of the genes in $H_{g}^{B}$ is the ortholog to *g*? The Best Reciprocal Hits (BRH) method is commonly used to find orthologs, however, BRH assumes that ortholog *g_i_* is necessarily that which is most similar to *g*, discounting the possibility of different evolutionary rates of paralogs. We therefore take a different approach in determining ortholog identity for genes in homology blocks. When selecting a single ortholog among all possible homologs in *H_g_*, we use synteny and sequence similarity to determine which of the genes in an examined genome is the correct ortholog. To do so we use the following three criteria:

1. **Prioritizing by gene blocks** We prioritize orthologs that are in gene blocks over orthologs that are isolated in the genome, and we look for the minimal number of such blocks that contain a representative of every ortholog that we recover. Example: the operon in *E.coli* had gene block (*abcdef*). The target genome has the following orthologs grouped in its genome: (*abcd*), (*abc*). In this case, we will choose as orthologs the genes populating (*abcd*).
2. **Recovering maximum number of genes** We consider the number of genes found. Example: the reference taxon operon had the gene block (*abcdef*). The studied genome has the following blocks (*abcd*) and ((abc),(de)). We would choose ((abc),(de)), two blocks,even though (*abcde*) is one block, because in the latter case we recover more homologs.
3. **Minimizing duplications** If in the target genome we have a choice between ortholog groups ((abc),(de)) or ((abcd),(de)), we choose the first because it has the minimal number of gene duplications.

We now define a *target homolog* as a gene in the target genome that is a homolog to a gene in a gene block in the reference genome *E.coli*. and a *target homolog block* as one or more target homologs, spaced ≤500 bp.

1: **for** geneBlock in ReferenceGenome do
2:  **for** genes in geneBlock **do**
3:   Find all homologs in the target genome with BLAST e-value $\leq 10^{-10}$
4:  **end** **for**
5:  Find all homologs in the target genome that are neighboring (≤500 bp)
6:  Use a greedy algorithm to recover the maximum number of target homologs prioritizing by gene blocks while minimizing gene duplications and maximizing number of genes recovered.
7: **end** **for**

#### 3.2.2 Event-based distances

Once orthologs are chosen, we are able to define the event-based distance between any two gene blocks with respect to split events, duplication events and deletion events. The distance between any two homologous gene blocks found in target organisms is defined as follows:

1. *Split distance* (*d*_s_) is the absolute difference in the number of relevant gene blocks between the two taxa. Example: for the reference gene block with genes (*abcdefg*) Genome *A* has blocks ((abc),(defg)) and genome *B* has ((abc),(de),(fg)). Therefore, $d_{s}(A,B)=|2-3|=1$.
2. *Duplication distance* (*d*_u_) is the pairwise count of duplications between two orthoblocks. Example: we have a reference gene block (*abcde*). Now, for genomes A and B the orthoblocks are A=((abd)) B=((abbcc)). Gene *A*_b_ is duplicated in genome *B*, thus a duplication distance $d_{u}(A,B)$ of 1. Gene *c* generates a distance of one deletion (see below) and one duplication. This is because the most parsimonious explanation is that the most recent ancestor for *A* and *B* may have had one copy of *c*, thus generating a duplication in one lineage, and a deletion in another. Because gene *d* exists only in the reference genome, it has no bearing on the event-based distance between the homologous gene blocks *A* and *B*.
3. *Deletion distance d*_d_ is the difference in number of orthologs that are in the homologous gene blocks of the genome of one organism, or the other, but not in both.

### 3.2.3 Gene block frequency matrices: phylomatrices

The rules outlined above allow us to determine the pairwise distance, for a given event and gene block, between any two genomes in our corpus. To visualize the frequency of an event in a block, we created matrices whose axes are the examined species, and whose cell is the normalized value of the pairwise distance for any given event. For an event *v* being one of insertion, deletion or duplication, for any two taxa *i* and *j* with homology blocks, the value for the normalized distance matrix entry *M_ij_* is:

$$M_{\text{ij}}=\frac{d_{\text{v}}(i,j)-{\bar{x}}_{d_{v}}}{\sigma_{d_{v}}}$$

where ${\bar{x}}_{d_{v}}$ is the mean value of the distance for event *v* calculated over all pairs of taxa *n*_p_ sharing that event:

$${\bar{x}}_{d_{v}}=\frac{1}{n_{\text{p}}}\sum_{i<j}d_{\text{v}}(i,j)$$

and $\sigma_{d_{v}}$ is the standard deviation.

### 3.2.4 Choice of proteobacteria species and phylogenetic tree construction

We chose our species as in Fani *et al.* (2005), removing a few species that we deemed to be too evolutionarily close. The phylogenetic trees shown in the study were constructed from multiple sequence alignments of the *rpoD* gene, using ClustalX 2.1 (Larkin *et al.,* 2007), followed by neighbor-joining. Supplementary Table S3 lists the species used.

## 4 Results

The results of this study show an interesting variety in gene block evolution. First, we show the *gene block tree* diagrams in a phylogenetic tree. These diagrams show the gene blocks as we find them in the different species we examine. The lack of a gene in the gene block phylogenetic tree does not mean the homolog does not exist in that taxon, but rather that it is no longer detectable by BLAST at the threshold of 10^−^^10^. Further, if a gene block is missing from the phylogenetic tree, it means that there are no two genes in the gene block that are neighboring in the genome within a distance of ≤500 bp.

**Fig. 2.**
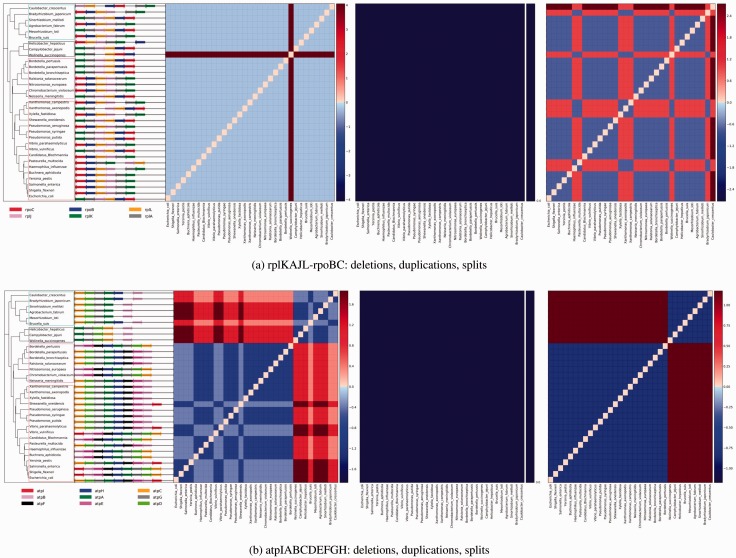
Highly conserved orthoblocks. The color matrices, which we named phylomatrices, each show the degree of relative conservation of the event between any two species. Left to right: Deletions, duplications, splits. Blue to red scale is high-to-low conservation *z*-score as described in Section 3. The boxes outline (top to bottom): *α*-, ϵ-, *β*-, and *γ*-proteobacteria. (**a**) rplKAJL-rpoBC has only a single gene deletion in *Wollinela*, no gene duplications, and a few splits (red squares, rightmost panel) including genes that moved to another strand. (**b**) the atp orthoblock shows deletions of *atpI* and a false deletion of *atpE* due to low similarity to *E.coli atpE* in ϵ and *α* proteobacteria (left matrix). No gene duplications are exhibited (middle panel). Splits are due to strand reversal of component genes (right panel. High-resolution figure available at: http://iddo-friedberg.net/operon-evolution/

**Fig. 3.**
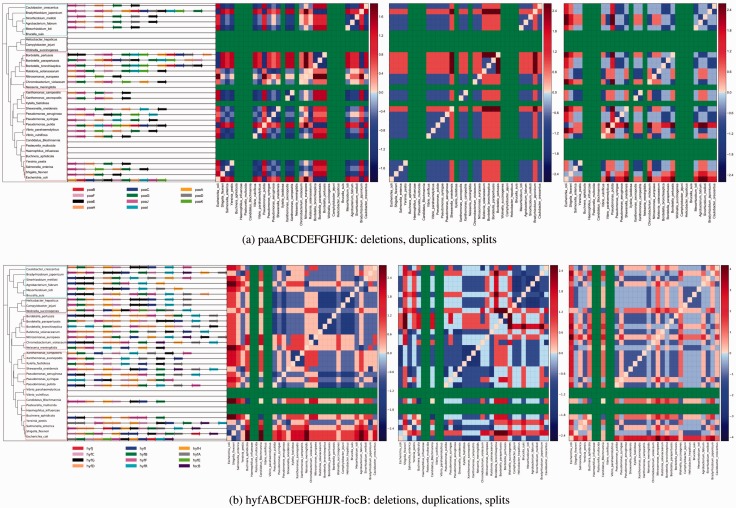
Phylomatrix diagrams of less conserved orthoblocks. Green squares are genomes in which component genes were not found using BLAST. (**a**) phenylacetate degradation orthoblock. (**b**) the hyf operon encoding the fourth hydrogenase in *E.coli* is not expressed under known conditions. See text for details on both operons. High-resolution figure available at: http://iddo-friedberg.net/operon-evolution/

To visualize the frequency of events, we generate gene block event frequency matrices or colored phylomatrices as described in Section 3. The value of each matrix is a *z*-score. Figure 2 shows two conserved gene blocks, and Figure 3 shows two non-conserved gene blocks.

### 4.1 Conservation of gene blocks and relationship to function

**Fig. 4.**
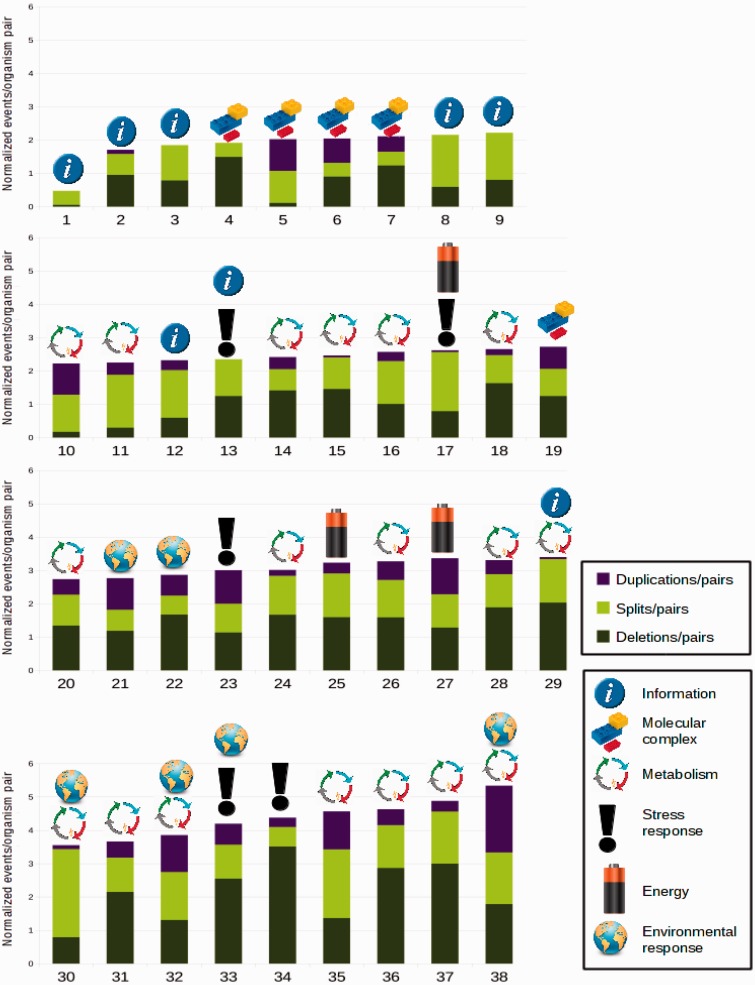
Relative conservation of operons and their primary biological functions. Each bar shows the cumulative number of events per genome pair per orthoblock. The orthoblocks are numbered as in Supplementary Table S1. Conserved orthoblocks have shorter bars, operons are ordered top-to-bottom left-to-right from most conserved to least conserved

The event-driven method enables us to examine the relative conservation of gene blocks in proteobacteria. Figure 4 the gene blocks are arranged in descending order of conservation. The most conserved block is the operon rplKAJL-rpoBC, a highly conserved transcription unit of ribosomal proteins (*rplK,* *rplK,* *rplA* and *rplL*) and two RNA polymerase subunits (*rpoB* and *rpoC*) (Steward and Linn, 1991), Figure 2(a).

No gene duplications or deletions were detected. Our program does erroneously call a deletion of the rplJ in *Wolinella*, but this is an error due to the stringent *e*-value cutoff and having a single exemplar of the *rplJ* gene as a query. The splits we detect are mostly between the *rplKA* and *rplJL-rpoBC* transcription units. The genes in this operon have multiple promotor and attenuator sites (Ralling and Linn, 1984), and have been shown to be governed by a complex set of signals (Steward and Linn, 1991). It appears that a complete tetracistronic product is transcribed from rplKAJL with less abundant bicistronic products of *rplK-rplA* and *rplJ-rplL* (Downing and Dennis, 1987), which may explain the strong conservation of these four genes in this gene block.

Another well-conserved operon is the atp operon, which codes for the genes for the *H*^+^-ATPase complex. ATP synthase is responsible for generating ATP using the proton motive force across the cell membrane (Fernandez Moran *et al.,* 1964). We examined the operon coding for ATP synthase, atpIBEFHAGDC. Although highly conserved, this operon does exhibit gene deletions in some taxa. Most notably the gene atpI, a nonessential gene that codes for a helper protein that assists the assembly of the ATP-synthase complex’s rotor. We readily recover this gene in orthoblocks in organisms that are closely related to *E.coli*. We did not observe any duplications in our dataset, The atpI deletion was a true deletion, which makes sense functionally as the *atpI* gene codes for the AtpI protein which is a nonessential component of the *H*^+^-ATPase complex. The other components supposedly deleted, *afpF,* *atpE* in ϵ-proteobacteria and *α*-proteobacteria are highly dissimilar to the equivalent *E.coli* genes, and are therefore not identifiable as homologs (*e*-value using BLAST > 0.01, data not shown; Figure 2b).

At the other edge of the conservation spectrum, we examined the hyf operon (Andrews *et al.,* 1997). As the phylomatrix shows, the gene block of 12 genes is not conserved as a single block in any of the proteobacteria in our data set. The hyf proton-translocating formate hydrogen lyase block of 12 genes appears to be an operon only in *E.coli*, although many of the genes appear in separate blocks in other bacteria. The hyf operon in *E.coli* is probably silent, at least under the environmental conditions examined, and has only been expressed under artificial conditions (Self *et al.,* 2004). Not being able to express it in *E.coli* under native conditions suggests it may be redundant, as does its lack of conservation in the species examined. See Figure 3(a).

The paa operon in *E.coli* encodes for a multicomponent oxygenase/reductase subunit for the aerobic degradation of phenylacetic acid. The *E.coli* operon comprises 11 genes. The distribution of the genes of this operon in 102 bacterial genomes was studied in detail in (Martin and McInerney, 2009). The authors’ conclusions from this study were that *de novo* clustering of some of this orthoblock’s genes occur repeatedly, due to weak selective pressure. The proximity of genes sets up opportunities for co-transcription. Specifically, this study has shown that genes *paaA,* *B,* *C* and *paaD*, when they are found, always co-occur in an operon. This makes sense, as those genes form a stable molecular complex with those genes coding for essential subunits for the degradation of phenylacetate (Grishin *et al.,* 2011). Both our study and Martin *et al*.’s (2009) found the full gene block only in *E.coli* and *Pseudomonas putida*. Another gene-block co-occurrence we find in our study is that of paaF and paaG, in 12 out of 23 species in which any components of the paa orthoblock occur. The products of these two genes form the FaaGH complex, another stable complex which catalyzes consecutive steps in the phenylacetate degradation pathway, and it was hypothesized that the proximity of the two proteins in a complex provides a fitness advantage (Grishin *et al.,* 2012).

Another use of the event-driven method is the reconstruction of ancestral gene blocks along the evolutionary tree. Supplementary Figure S1 shows such a reconstruction for paa in the *γ*-proteobacteria species used in our study. We manually examined the possible events needed to transition between the tree’s nodes, and along each branch minimized the number of events leading to the extant orthoblocks. One interesting outcome of this analysis, is that paa orthoblock appears to be the result of HGT events in *E.coli* and in *P.putida*, where an entire gene block exists in both species, but not in the closely related ones.

To determine if there is a relationship between gene block conservation and the function of the gene blocks, we assigned each operon keywords based on its function. The categories we used were Metabolism, Information, Molecular Complex, Stress Response, Energy and Environmental Response. The keywords were assigned based on reading the literature relevant for each operon, and the information provided in EcoCyc (Keseler *et al.,* 2013). As can be seen in Figure 4, from the gene blocks we studied, gene blocks whose function is information or protein complex tend to be more conserved which agrees with previous studies, and those dealing with stress response are less conserved. Gene blocks whose primary function was identified as metabolism were found at all levels of conservation. We conclude that there may be a relationship between gene block conservation and function, and that gene blocks having to do with information or molecular complexes are more conserved than those dealing with stress response and/or environmental response. Other studies have shown similar trends (Dandekar *et al.,* 1998).

## 5 Discussion and conclusions

We introduce a method to examine gene block and operon evolution in bacteria. This method, coupled with the phylomatrix visualization we introduce, enables the interrogation of the evolution of gene blocks in a bacterial clade. The event-driven approach we use allows for the quantification of evolutionary conservation of any gene block. Most importantly, the event-driven method does not attempt to present a predictive evolutionary model such as the models reviewed in the introduction. Rather, it allows for these models to be examined in specific gene blocks with specific taxa. The event-driven method is agnostic to any model predicting how an operon or gene block evolved.

To determine the conservation of gene blocks, a choice needs to be made for the proper orthologs between genomes. Identifying orthologs is a challenging problem that has been studied extensively. Several methods have been developed to do so including use of inter-species clusters (Powell *et al.,* 2014; Remm *et al.,* 2001; Tatusov *et al.,* 1997), reciprocal best hits (Tatusov *et al.,* 1997) or phylogenetic methods (Fulton *et al.,* 2006). Here we used genomic context and strict similarity criteria to choose which genes are orthologous, and are likely to have the same function. The ortholog choice method we use here assumes that evidence for orthology is strengthened by genomic context. This assumption has been shown to be useful for a more precise assignment of orthologs (Jun *et al.,* 2009; Ward and Moreno-Hagelsieb, 2014) and has been implemented in computational resources (Aziz *et al.,* 2008; Overbeek *et al.,* 2014; Szklarczyk *et al.,* 2014) to resolve ortholog ambiguities.

We choose gene blocks on the basis that, at least in one species (*E.coli*), the genes are co-transcribed. The initial motivation for this study was the observation that orthoblocks have been shown to be useful in inferring common function, even when co-transcription has not been determined (Enault *et al.,* 2005; Marcotte *et al.,* 1999; Nitschké *et al.,* 1998; Overbeek *et al.,* 1999). We have shown that tracking gene block evolution in bacteria through the tallying of simple events provides an objective method for quantifying their evolutionary conservation, relative to a reference species. Additionally, we have shown detailed examples of two highly conserved orthoblocks (atp and ydc) and two less-conserved orthoblocks (paa and hyf). Finally, we have related overall conservation to the type of orthoblock function, although a larger survey of orthoblocks is needed to obtain a more reliable picture.

## Funding

This work is supported, in part, by the National Science Foundation under (ABI-1146960). Any opinions, findings, and conclusions or recommendations expressed in this material are those of the authors and do not necessarily reflect the views of the National Science Foundation. Publication charges were provided, in part, by a Miami University Publication, Reprint, Exhibition, and Performance award given to IF.

*Conflict of Interest*: none declared.

## Supplementary Material

Supplementary Data
