# Supplementary material for: An event-driven approach for studying gene block evolution in bacteria
Source: Bioinformatics. 2015 Feb 25;31(13):2075–83. doi: 10.1093/bioinformatics/btv128 (PMC4481853; doi:10.1093/bioinformatics/btv128)
Supplement: Supplementary Data [file supp_31_13_2075__index.html]

An Event-Driven Approach for Studying Gene Block Evolution in Bacteria — An Event-Driven Approach for Studying Gene Block Evolution in Bacteria — An Event-Driven Approach for Studying Gene Block Evolution in Bacteria — An event-driven approach for studying gene block evolution in bacteria — An event-driven approach for studying gene block evolution in bacteria — Supplementary Data 

# An event-driven approach for studying gene block evolution in bacteria

## Supplementary Data

files

**Files in this Data Supplement:**

- Supplementary Data - pdf file
- Supplementary Data - pdf file
