# Supplementary material for: An event-driven approach for studying gene block evolution in bacteria
Source: Bioinformatics. 2015 Feb 25;31(13):2075–83. doi: 10.1093/bioinformatics/btv128 (PMC4481853; doi:10.1093/bioinformatics/btv128)
Supplement: Supplementary Data [file supp_btv128_operon-sup.pdf]

Supplementary material to *An Event-Driven Approach for Studying Gene Block Evolution in Bacteria*

David C. Ream Asma R. Bankapur Iddo Friedberg

February 13, 2015

1 Online materials

:

1. Data matrices in tab-delimited format for all operons in this study are available at:
`http://iddo-friedberg.net/operon-evolution`

2. Heatmap images of data matrices for all operons in this study are available at:
`http://iddo-friedberg.net/operon-evolution` (large, 68GB file)

3. High-resolution versions of Figures 1 & 2 from the main text are available at:
`http://iddo-friedberg.net/operon-evolution`

4. Software and additional data are available at:
`http://github.com/reamdc1/gene_block_evolution.git`

2 Supplementary Tables

num	Operon	Dels	Splits	Dups	Species	Sum/species	Sum/pairs
1	rplKAJL-rpoBC	34	250	0	35	8.11	0.48
2	ybgIJKL-nei	115	75	15	16	12.81	1.71
3	rnc-era-recO-pdxJ-acpS	391	526	0	32	28.66	1.85
4	atpIBEFHAGDC	890	250	0	35	32.57	1.92
5	mdtABCD-baeSR	62	506	502	33	32.42	2.03
6	pstSCAB-phoU	478	216	386	33	32.73	2.05
7	livKHMGF	504	166	188	29	29.59	2.11
8	thrS-infC-rpmI-rplT-pheMST-ihfA	354	928	0	35	36.63	2.15
9	lptD-surA-pdxA-rsmA-apaGH	376	656	0	31	33.29	2.22
10	trpLEDCBA	99	622	526	34	36.68	2.22
11	hisLGDCBHAFI	168	888	208	34	37.18	2.25
12	metY-rimP-nusA-infB-rbfA-truB-rpsO-pnp	336	799	168	34	38.32	2.32
13	nsrR-rnr-rlmB-yjfIJ	262	232	0	21	23.52	2.35
14	astCADBE	217	97	56	18	20.56	2.42
15	sdhCDAB-sucABCD	870	562	34	35	41.89	2.46
16	yceD-rpmF-plsX-fabHDG-acpP-fabF	600	764	168	35	43.77	2.57
17	bamA-hlpA-lpxD-fabZ-lpxAB-rnhB-dnaE	472	1054	34	35	44.57	2.62
18	fucPIKUR	90	46	10	11	13.27	2.65
19	nuoABCEFGHIJKLMN	580	378	310	31	40.90	2.73
20	ivbL-ilvBN-uhpABC	626	430	218	31	41.10	2.74
21	fecABCDE	628	332	502	33	44.30	2.77
22	ssuEADCB	830	283	310	32	44.47	2.87
23	hcaEFCBD	120	90	106	15	21.07	3.01
24	gcl-hyi-glxR-ybbVW-allB-ybbY-glxK	92	64	10	11	15.09	3.02
25	hypABCDE-fhlA	88	72	18	11	16.18	3.24
26	rbsDACBKR	694	486	245	30	47.50	3.28
27	hycABCDEFGHI	600	462	504	31	50.52	3.37
28	glcDEFGBA	324	170	82	19	30.32	3.37
29	lptAB-rpoN-hpf-ptsN-yhbJ-npr	1210	780	34	35	57.83	3.40
30	yjeFE-amiB-mutL-miaA-hfq-hflXKC	394	1310	60	32	55.13	3.56
31	srlAEBD-gutM-srlR-gutQ	142	68	32	12	20.17	3.67
32	caiTABCDE	394	430	334	25	46.32	3.86
33	lsrACDBFG-tam	1110	441	277	30	60.93	4.20
34	waaQGP-rfaS-waaBIJY-rfaZ-waaK	320	53	26	14	28.50	4.38
35	tdcABCDEFG	144	216	120	15	32.00	4.57
36	paaABCDEFGHIJK	792	355	132	24	53.29	4.63
37	yiaKLMNO-lyxK-sgbHUE	760	394	82	23	53.74	4.89
38	hyfABCDEFGHIJR-focB	832	720	932	31	80.13	5.34

Table 1: Gene blocks ranked by conservation. Dels: total deletion count; Splits: total pairwise count; Dups: total duplications counts. Species: number of species in which the orthoblock was found.

NCBI ID	Species name
NC_000913	*Escherichia coli*
NC_004741	*Shigella flexneri*
NC_003197	*Salmonella enterica*
NC_003143	*Yersinia pestis*
NC_002663	*Pasteurella multocida*
NC_004459	*Vibrio vulnificus*
NC_004603	*Vibrio parahaemolyticus*
NC_011833	*Buchnera aphidicola*
NC_000907	*Haemophilus influenzae*
NC_004347	*Shewanella oneidensis*
NC_005061	*Candidatus Blochmannia*
NC_002516	*Pseudomonas aeruginosa*
NC_002947	*Pseudomonas putida*
NC_004578	*Pseudomonas syringae*
NC_003919	*Xanthomonas axonopodis*
NC_007086	*Xanthomonas campestris*
NC_002488	*Xylella fastidiosa*
NC_004757	*Nitrosomonas europaea*
NC_003295	*Ralstonia solanacearum*
NC_005085	*Chromobacterium violaceum*
NC_003112	*Neisseria meningitidis*
NC_002927	*Bordetella bronchiseptica*
NC_002929	*Bordetella pertussis*
NC_002928	*Bordetella parapertussis*
NC_004463	*Bradyrhizobium japonicum*
NC_002696	*Caulobacter crescentus*
NC_003062	*Agrobacterium fabrium*
NC_002678	*Mesorhizobium loti*
NC_003047	*Sinorhizobium meliloti*
NC_004310	*Brucella suis*
NC_005090	*Wolinella succinogenes*
NC_004917	*Helicobacter hepaticus*
NC_002163	*Campylobacter jejuni*

Table 2: NCBI ID's and species names for genomes used in this study

**Title**	**Definition**
Information	Involved in the transfer of information: transcription, translation or signaling.
Molecular complex	The gene products assemble, forming a molecular complex.
Metabolism	Having to do with the assembly or breakdown of compounds, excluding energy.
Stress response	Gene products respond to cold, heat, water, salinity, pH, and other stress-related changes.
Energy	Respiration or other forms of metabolic energy production.
Environmental response	Responding to changes in environment, not stress-related.

Table 3: Definitions of operon functions used in Figure 4

Supplementary Figure 1

Here we show an ancestral reconstruction of the paaABCDEFGHIJK operon, using the12 γ-proteobacteria in the study.The phylogenetic tree used is the species tree, constructed as described in Methods. The following considerations were applied: for each two child orthoblocks, a putative parent with a distance of one possible event from either child was enumerated. As the inner nodes were filled right-to-left, the less-probable parents (those requiring a larger number of events to transition to a child) were removed. If at the end of the process two more than one possible parent remained, one was selected arbitrarily and the process repeated until only a single orthoblock remained in each node. For two species, *E. coli* and *P. putida* it was impossible to find close ancestors explaining them and the species near them. We therefore hypothesize horizontal gene transfer events for these two species.
