# Supplementary figures and images for: An event-driven approach for studying gene block evolution in bacteria
Source: Bioinformatics. 2015 Feb 25;31(13):2075–83. doi: 10.1093/bioinformatics/btv128 (PMC4481853; doi:10.1093/bioinformatics/btv128)

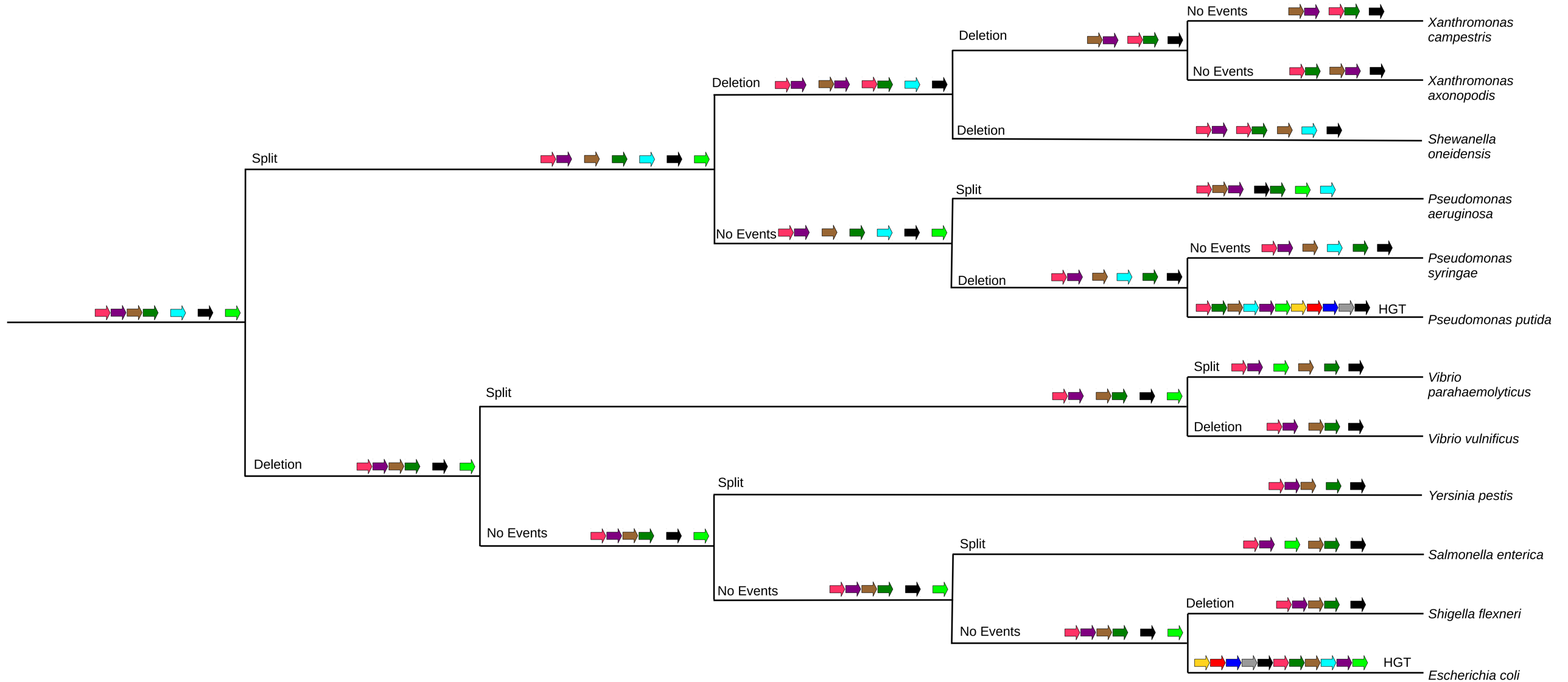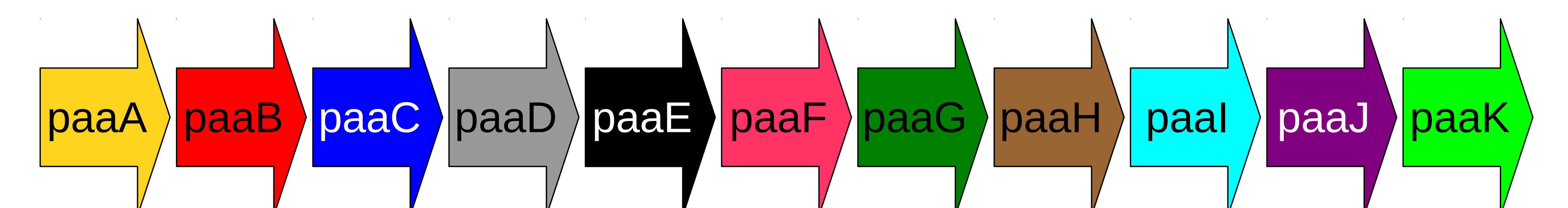

Supplement: Supplementary Data [file supp_btv128_sup-fig1.pdf]
